# Harmonisation and Between-Country Differences of the Lifetime of Experiences Questionnaire in Older Adults

**DOI:** 10.3389/fnagi.2021.740005

**Published:** 2021-10-14

**Authors:** Valentin Ourry, Natalie L. Marchant, Ann-Katrin Schild, Nina Coll-Padros, Olga M. Klimecki, Pierre Krolak-Salmon, Karine Goldet, Leslie Reyrolle, Romain Bachelet, Lena Sannemann, Dix Meiberth, Harriet Demnitz-King, Tim Whitfield, Maëlle Botton, Julie Lebahar, Julie Gonneaud, Robin de Flores, José Luis Molinuevo, Frank Jessen, Denis Vivien, Vincent de la Sayette, Michael J. Valenzuela, Géraldine Rauchs, Miranka Wirth, Gaël Chételat, Eider M. Arenaza-Urquijo, The Medit-Ageing Research Group

**Affiliations:** ^1^Normandie University, UNICAEN, INSERM, U1237, PhIND “Physiopathology and Imaging of Neurological Disorders”, Institut Blood and Brain @ Caen-Normandie, Caen, France; ^2^Normandie University, UNICAEN, PSL Université, EPHE, INSERM, U1077, CHU de Caen, GIP Cyceron, NIMH, Caen, France; ^3^Division of Psychiatry, University College London, London, United Kingdom; ^4^Department of Psychiatry, Medical Faculty, University of Cologne, Cologne, Germany; ^5^Alzheimer's Disease and Other Cognitive Disorders Unit, Hospital Clinic, IDIBAPS, Barcelona, Spain; ^6^Clinical Psychology and Behavioural Neuroscience, Technische Universität Dresden, Dresden, Germany; ^7^Clinical and Research Memory Center, Hospices Civils de Lyon, Université de Lyon, INSERM, Lyon, France; ^8^Hospices Civils de Lyon, Institut du Vieillissement, CRC Vieillissement-Cerveau-Fragilite, Lyon, France; ^9^Département de Recherche Clinique, CHU Caen-Normandie, Caen, France; ^10^Service de Neurologie, CHU de Caen, Caen, France; ^11^School of Psychiatry, University of New South Wales, Sydney, NSW, Australia; ^12^Skin2Neuron Pty Ltd., Sydney, NSW, Australia; ^13^German Centre for Neurodegenerative Diseases (DZNE), Dresden, Germany; ^14^Barcelonabeta Brain Research Center, Fundación Pasqual Maragall, Barcelona, Spain

**Keywords:** cognitive reserve, lifetime of experiences questionnaire, life-course, older adults, harmonisation, cross-cultural validation

## Abstract

**Background:** The Lifetime of Experiences Questionnaire (LEQ) assesses complex mental activity across the life-course and has been associated with brain and cognitive health. The different education systems and occupation classifications across countries represent a challenge for international comparisons. The objectives of this study were four-fold: to adapt and harmonise the LEQ across four European countries, assess its validity across countries, explore its association with brain and cognition and begin to investigate between-country differences in life-course mental activities.

**Method:** The LEQ was administered to 359 cognitively unimpaired older adults (mean age and education: 71.2, 13.2 years) from IMAP and EU-funded Medit-Ageing projects. Education systems, classification of occupations and scoring guidelines were adapted to allow comparisons between France, Germany, Spain and United Kingdom. We assessed the LEQ's (i) concurrent validity with a similar instrument (cognitive activities questionnaire - CAQ) and its structural validity by testing the factors' structure across countries, (ii) we investigated its association with cognition and neuroimaging, and (iii) compared its scores between countries.

**Results:** The LEQ showed moderate to strong positive associations with the CAQ and revealed a stable multidimensional structure across countries that was similar to the original LEQ. The LEQ was positively associated with global cognition. Between-country differences were observed in leisure activities across the life-course.

**Conclusions:** The LEQ is a promising tool for assessing the multidimensional construct of cognitive reserve and can be used to measure socio-behavioural determinants of cognitive reserve in older adults across countries. Longitudinal studies are warranted to test further its clinical utility.

## Introduction

Cognitive Reserve (CR) refers to “the adaptability (i.e., efficiency, capacity, flexibility) of cognitive processes that helps to explain differential susceptibility of cognitive abilities or day-to-day function to brain ageing, pathology, or insult” (Stern et al., 2018). Cognitive reserve is determined by environmental and genetic factors (Valenzuela, 2019), nurtured throughout the life-course by exposure to mentally stimulatory activities. These include, but are not limited to, education (Stern et al., 1992), occupation complexity (Stern et al., 1995) and leisure activities (Scarmeas et al., 2001), sometimes referred to as “CR proxies.” Several studies support the finding that higher exposure to such proxies is associated with lower dementia incidence and with a significant reduction of cognitive decline (Valenzuela and Sachdev, 2006a,b; Xu et al., 2019). Furthermore, life-course mental activity may act as a protective factor against Alzheimer's disease (AD), both at early and later stages of the continuum, *via* different brain mechanisms (Arenaza-Urquijo et al., 2015).

The use of CR proxies in research can be limited, especially when they are used separately or evaluated during a specific period, as they may not reflect the full exposure to stimulating activities during the life-course. In order to address this, several complex psychometric instruments were proposed to measure the construct of CR (Wilson et al., 2003; Rami et al., 2011; León et al., 2014) including the Lifetime of Experiences Questionnaire (LEQ) (Valenzuela and Sachdev, 2007). The LEQ is a multidimensional instrument assessing complex mental activity, including education, occupation complexity and leisure activities, across the life-course (Valenzuela and Sachdev, 2007). The original LEQ version (Australian version) has been validated in cognitively normal older adults and has demonstrated good clinical validity; for instance it has predicted cognitive decline over 18 months (Valenzuela and Sachdev, 2007) and rate of hippocampal atrophy (Valenzuela et al., 2008). Furthermore, its low cost and easy administration are an advantage for research projects investigating reserve related-mechanisms. Therefore, the LEQ appears to be a promising tool to capture CR (Kartschmit et al., 2019). However, cross-cultural adaptation is pending (Landenberger et al., 2019). Further, the LEQ needs to be translated and the education and occupation systems harmonised so that it can be used for international comparisons.

This study was carried out in the framework of a European project where we aimed at assessing lifestyle cognitive activity across five centres in four different countries. The objectives of this study were first to harmonise and translate the LEQ, and as follows: (i) to evaluate concurrent and structural validity of the adapted LEQ by assessing its correlation with a similar tool and its factor's structure across four European countries, respectively, (ii) to test associations with cognitive domains affected in ageing and AD, and brain measures previously associated with the LEQ or other CR proxies (Valenzuela et al., 2008; Arenaza-Urquijo et al., 2013), and (iii) to assess between-country differences in life-course mental activities.

## Materials and Methods

### Participants

This cross-sectional study included 359 cognitively unimpaired older adults, 46.2% of whom met criteria for subjective cognitive decline (SCD) as proposed by the international working group for SCD (SCD-I; Jessen et al., 2014) (see flow diagram in Supplementary Figure 1B). Participants were recruited from the community or from medical facilities (e.g., memory clinics) in five European centres [Caen and Lyon, France; Cologne, Germany; Barcelona, Spain; London, United Kingdom (UK)] and were part of the Medit-Ageing European project (Age-Well, Poisnel et al., 2018 and SCD-Well, Marchant et al., 2018) or the multimodal imaging of Alzheimer's disease at an early stage project (IMAP) (La Joie et al., 2012; Arenaza-Urquijo et al., 2016; Perrotin et al., 2017; Kuhn et al., 2019). Specific eligibility criteria for each protocol are detailed elsewhere (La Joie et al., 2012; Marchant et al., 2018; Poisnel et al., 2018). Both the Medit-Ageing and IMAP projects have included SCD participants which explains the high proportion of SCD participants in this study. Briefly, all participants were aged over 60 years and performed within the normal range on standardised cognitive tests. They all underwent lifestyle evaluation. Age-Well participants (*n* = 135) also underwent neuropsychological and neuroimaging evaluation. All participants provided their written informed consent and each study was approved by the local ethics committee. Participant's characteristics are presented in Table 1.

**Table 1:** Country description.

**Measure**	**France** **(*n* = 252)**	**Germany** **(*n* = 39)**	**Spain** **(*n* = 40)**	**United Kingdom** **(*n* = 28)**	**Total** **(*n* = 359)**	**Group comparison**		**Pairwise comparisons**
						***F-* and *P*-value**	**Effect size**	
Age (years)	70.8 ± 6 (66.5, 69.5, 74.2)	71.8 ± 6.3 (67, 72.1, 76.1)	71.7 ± 5.5 (67.8, 72, 76.1)	74 ± 7.2 (68.6, 73.3, 78.8)	71.3 ± 6.1 (66.8, 69.8, 75.3)	*F*_(3,355)_ = 2.42 *P* = 0.066		
Female: *n* (%)	159 (63.1%)	22 (56.4%)	26 (65%)	9 (32.1%)	216 (60.2%)	Fisher's exact test *P* = 0.015	Cramér's *V* = 0.173	FR > UK^*^, SP > UK^*^
Education (years)	13 ± 3.5 (10, 14, 15)	14.2 ± 2.9 (12, 14, 16)	13.8 ± 3.9 (11, 15, 17)	12.8 ± 3.3 (11, 11, 15)	13.2 ± 3.5 (10, 14, 16)	*F*_(3,355)_ = 1.8 *P* = 0.147		
MMSE	28.9 ± 1.1 (28, 29, 30)	29.4 ± 0.8 (29, 30, 30)	28.9 ± 1.2 (28, 29, 30)	28.2 ± 1.1 (27, 28, 29)	28.9 ± 1.1 (28, 29, 30)	*F*_(3,355)_ = 7.32 *P* < 0.001	η^2^ = 0.058	FR^**^-SP^*^- GER^**^> UK, GER > FR^*^
Total LEQ	97.4 ± 19.6 (84, 96.8, 110.5)	104.9 ± 19.8 (89.9, 105.2, 119.3)	106.7 ± 24.7 (84.9, 109.4, 124)	99.7 ± 19.4 (82.8, 100.2, 110.8)	99.4 ± 20.5 (84, 98.8, 112.9)	*F*_(3,332)_ = 3.36 *P* = 0.019	η^2^ = 0.029	SP > FR^*^
Total CAQ	3.1 ± 0.6 (2.7, 3.2, 3.5)	3.2 ± 0.5 (2.8, 3.2, 3.7)	2.9 ± 0.7 (2.4, 2.9, 3.3)	3 ± 0.5 (2.5, 3, 3.4)	3.1 ± 0.6 (2.7, 3.1, 3.5)	*F*_(3,344)_ = 2.85 *P* = 0.04	η^2^ = 0.024	GER > SP^t^

*LEQ, Lifetime of Experiences Questionnaire; CAQ, Cognitive Activities Questionnaire; MMSE, Mini Mental State Examination; FR, France; GER, Germany; SP, Spain; UK, United Kingdom*.

t *P < 0.1*,

* *P < 0.05*,

** *P < 0.01*,

^***^ P < 0.001.

*For numerical variables, the mean ± standard deviation (first quartile, median, and third quartile) are indicated. Tukey's Honestly Significant Difference test was used as a post-hoc test. For sex proportion differences, Fisher's exact test was used*.

### The Lifetime of Experiences Questionnaire (LEQ)

The adapted and harmonised version of the LEQ (see section Between-country translation and Harmonisation for harmonisation procedure) was self-completed by participants and checked by a neuropsychologist or a psychometrist to minimise missing data. When missing values were identified the information was collected *via* phone calls. This questionnaire assesses complex mental activity (e.g., education, occupational complexity, cognitive activities) across three life periods: young adulthood (13–30 years), mid-life (30–65 years), and late life (from 65 years to present date) (Valenzuela and Sachdev, 2007), visually represented in Supplementary Figure 1A. Briefly, each life period comprises two scores (specific and non-specific, see below) which were summed to give three sub-scores of the LEQ (young adulthood, mid-life and late life). Then, these three sub-scores can be summed to create a LEQ total score where higher scores reflect higher levels of complex mental activity across the life-course.

#### Specific Sub-scores of the LEQ

The young adulthood specific score was calculated using years spent in primary and secondary school education which were harmonised across countries (see next section and Supplementary Table 1), as well as post-secondary school education (Valenzuela and Sachdev, 2007).

The mid-life specific score was calculated based on occupation types which were harmonised across countries (see next section and Supplementary Table 2), managerial experience and additional education during this period (Valenzuela and Sachdev, 2007).

The late life specific score was calculated based on social, leisure and information-seeking (e.g., How do you usually acquire your information about world and national events?) behaviours, and additional education during this period (Valenzuela and Sachdev, 2007).

The specific scores were normalised so that they have the same weighting for each period (Valenzuela and Sachdev, 2007).

#### Non-specific Sub-scores of the LEQ

The content of non-specific scores were similar across all life periods and were computed exactly as in the original LEQ (Valenzuela and Sachdev, 2007). In brief, the non-specific score included a standard set of seven questions about the participation in various leisure activities such as visits to family or friends, music practise, participation in artistic activities (e.g., drawing, painting, writing), physical activities (of mild, moderate or high intensity), reading, second language practise and travelling. Each question was scored from 0 to 5, and summed to produce a theoretical maximum score of 35.

### Between-Country Translation and Harmonisation

The LEQ was first translated from English by a professional translation service (Alphatrad United Kingdom: www.alphatrad.com; Certificate number GBO 0/1238) and iteratively edited by native speakers for Spain and Germany who were also fluent in English. A French version of the LEQ was already available (C Berr & D Villebrun, INSERM U1061, Montpellier, France) and has been used with slight changes of format and harmonisation (see below).

The adaptations made to harmonise the LEQ across countries concerned education and occupation items, and the scoring guidelines. Former education systems differed in the duration of the educational stages (primary and secondary school) (Supplementary Table 3). Taking into account between-country differences in primary and secondary school duration, we gave the maximum score (+8) for the last 2 years of secondary school, followed by the previous 3 years (+4) and 1 year or less of secondary school (+0) (except for France where 2 years were scored as +0 because there is one additional year of secondary school) (Supplementary Table 1). Concerning the scoring of occupations, the original version of the LEQ used a national guide, the Australian Standard Classification of Occupations (Australian Bureau of Statistics, 1997). Therefore, here we changed it to an international guide, the International Standard Classification of Occupations (International Labour Office, 2008), that allowed comparability across European countries (Supplementary Table 2).

The material (LEQ) is available upon request (www.silversantestudy.eu).

### Cognitive Activities Questionnaire (CAQ)

Participants completed the Cognitive Activities Questionnaire (CAQ) which is a self-reported questionnaire assessing cognitive activities at different periods of the participant's life: childhood (at ages 6 and 12), transition to adulthood (at 18 years), middle of adulthood (at 40 years), and for the current period (Wilson et al., 2003). Responses were provided on a 5-point frequency scale ranging from 1 (once a year or less) to 5 (every day or almost every day). For each participant, we calculated three sub-scores: early life (average over the age periods 6, 12, and 18 y), mid-life (average of the age period 40 y) and late life (average of the current period), and a lifetime cognitive activity score (average over all age periods). These different scores are hereafter referred to as CAQ early life, CAQ mid-life, CAQ late life and total CAQ (Wirth et al., 2014). Both total LEQ and CAQ are a reflect of cognitively stimulating experiences across the life-course (Wilson et al., 2003; Valenzuela and Sachdev, 2007). However, the CAQ evaluates leisure activities but not education or occupational attainments (reflected in the LEQ specific sub-scores). Thus, the CAQ questions are closer to the non-specific questions of the LEQ (see above).

The CAQ was translated from English by a professional translation service (Alphatrad United Kingdom: www.alphatrad.com; Certificate number GBO 0/1238) and iteratively edited by native speakers for France, Spain, and Germany who were also fluent in English. The CAQ–a measure that has been previously validated–focuses on relatively common activities with minimal physical demands or social requirements to enhance the applicability of the scale among persons with diverse cultural and socioeconomic backgrounds (Wilson et al., 2003).

### Neuroimaging Data

#### Data Acquisition

Participants from the Age well-randomised controlled trial only were scanned at the Cyceron Centre (Caen, France) on the same MRI (Philips Achievia 3.0T scanner). A high-resolution T1-weighted anatomical image using a three-dimensional fast-field echo sequence (Sagittal 3D-FFE, TR/TE = 7.1/3.3 ms, FOV = 256 × 256 mm^2^, 180 slices, 1 × 1 × 1 mm^3^) and a three-dimensional fluid-attenuated inversion recovery (FLAIR; sagittal; repetition time, 4,800 ms; echo time, 272 ms; inversion time, 1,650 ms; field of view, 250 × 250 mm2, 180 slices, voxel size: 0.98 × 0.98 × 1 mm^3^) were acquired.

#### Data Pre-processing

T1-weighted images were segmented using the Statistical Parametric Mapping (SPM12) software's multiple channels segmentation procedure (http://www.fil.ion.ucl.ac.uk/spm/software/spm12) with the corresponding FLAIR images. Grey matter (GM) segments were spatially normalised to the Montreal Neurological Institute (MNI) template and modulated, correcting for the effects of non-linear warping but not affine transformation in order to take into account brain size variation. The anterior cingulate cortex GM volume values were obtained by applying the corresponding binary mask from the Harvard-Oxford cortical/subcortical atlases (Desikan et al., 2006; Makris et al., 2006) on the pre-processed GM maps.

The anterior and posterior hippocampus were automatically segmented from T1-weighted images using the Automatic Segmentation of Hippocampal Subfield (ASHS) software (Xie et al., 2019) with the ASHS-T1 pipeline (https://sites.google.com/view/ashs-dox/home). Segmentations were visually inspected and corrected in case of error. Total Intracranial Volume (TIV) was measured using SPM12. Hippocampal volume values automatically obtained during the processing step with the ASHS-T1 pipeline were corrected for TIV and referred to as the average volume of left and right hippocampus (anterior plus posterior segments).

### Neuropsychological Data

Participants from the Age well-randomised controlled trial only underwent the following neuropsychological tests: the Dementia Rating Scale−2 (DRS-2, Jurica et al., 2001), the Wechsler Adult Intelligence Scale Revised (WAIS-IV, Wechsler, 1981), the California Verbal Learning Test (CVLT-II, Delis et al., 2000), the Wechsler Memory Scale (WMS-III, Powel, 1988) and the Category fluency developed by a reflection group on executive functions evaluation (GREFEX).

The Pre-clinical Alzheimer's Cognitive Composite 5 (PACC5) is a global cognitive composite sensitive to detecting and tracking pre-clinical AD-related decline (Papp et al., 2017). To calculate the PACC5 in Age-Well, we first standardised the scores of each of its constituents: the Dementia Rating Scale-2 (total score), WAIS-IV Coding (raw score), CVLT-II (delayed recall score), WMS-III Logical Memory (delayed recall score), and Category Fluency (number of correct animals recalled in 2 min). We then took the unweighted average of these five *z*-scores, yielding the PACC5. A higher score reflects greater cognitive performance.

### Statistical Analyses

#### Concurrent and Structural Validity

*Concurrent Validity*. Concurrent validity was tested by whether the adapted and harmonised version of the LEQ correlated with the CAQ. Pearson correlations were performed, firstly between total LEQ and total CAQ scores, and secondly between each life period of the LEQ (non-specific young adulthood, mid-life and late life measuring leisure activities engagement) and the corresponding life period of the CAQ (early life, mid-life, and late life), both in the whole sample and within each country separately, using the *R* 4.0.2 software (R Core Team, 2020). Statistical significance was set at *p* < 0.05.

*Structural Validity*. Structural validity was assessed with a Dual Multiple Factor Analysis (DMFA) using the *R* 4.0.2 software (R Core Team, 2020) with the DMFA function from the *FactoMineR* package (Lê et al., 2008; Lê and Pagès, 2010).

DMFA is an extension of multiple factor analysis, using an internal principal components analysis, where data are partitioned in different sets (countries) with the same set of variables (LEQ items) (Abdi et al., 2013). Items data was first mean-centred and scaled using the standard deviation by country. Three items of the LEQ (i.e., social activity between 13 and30 years; social activity between 30 and 65 years and other education after 65 years) did not met criteria for DMFA as no standard deviation variation was detected in some countries. These items were therefore removed from the analysis so that the DMFA was conducted on the 38 remaining items of the LEQ.

A common eigenvalue of 1, corresponding to 1/38 = 2.6% variance explained, was used to identify dimensions (Kaiser, 1960). Both at the whole sample and country levels, we obtained (i) the scree plot of the percentage of variance explained by each dimension and (ii) the loading strength of each item (unrotated coordinates) on each dimension.

#### Association With Global Cognition and Regional Brain Volumes

To assess whether there was an association between the LEQ and global cognitive or brain measures, separate multiple linear regressions were performed using the *R* 4.0.2 software (R Core Team, 2020) in the French Age-well sample. The PACC5 or the hippocampal volume and the anterior cingulate cortex GM volume, brain areas that have previously shown associations with the LEQ or education (Valenzuela et al., 2008; Arenaza-Urquijo et al., 2013), were used as dependent variables, the total LEQ as independent variable and age and sex as covariates. Additionally, analyses were repeated with the young, mid and late sub-scores of the LEQ. Statistical significance was set at *p* < 0.05.

#### Between-Country Differences

Between-country differences for demographic and life-course mental activities were assessed using analyses of variances (ANOVAs). When statistical significance (*p* < 0.05) was reached, Tukey's Honestly Significant Difference tests were performed.

## Results

### Demographics and Total LEQ Scores

There were no between-country differences in participants' age (*p* = 0.07) or education (*p* = 0.15). Between-country differences were observed in the percentage of female participants (*p* = 0.02), with a lower proportion of women in UK (Table 1).

In the whole sample, the mean total LEQ was 99.4 ± 20.5, with a minimum of 50.6 and a maximum of 166.4. Total LEQ showed a normal Gaussian distribution (Shapiro test: *W* > 0.99, *p* > 0.05) suggesting no ceiling or floor effects. Between-country differences in the LEQ scores are detailed below in the corresponding section.

In the whole sample, the total LEQ was not associated with age (*p* = 0.56) and was positively associated with education (*p* < 0.001). Similar results were observed at the country-level: total LEQ was not associated with age (all *p* > 0.05) and was positively associated with education (*p* (UK) = 0.009; all other *p* < 0.001).

Furthermore, in the whole sample, between-sex differences were observed in total LEQ with men showing higher scores than women (*p* = 0.001). At country-level, between-sex differences were observed in total LEQ in Spain (*p* = 0.02) and UK (*p* = 0.006), with men showing higher scores than women. In Germany this was at a trend level (*p* = 0.06) and there was no significant difference in France (*p* = 0.19).

### Concurrent and Structural Validity

#### Concurrent Validity

**Figure 1:**
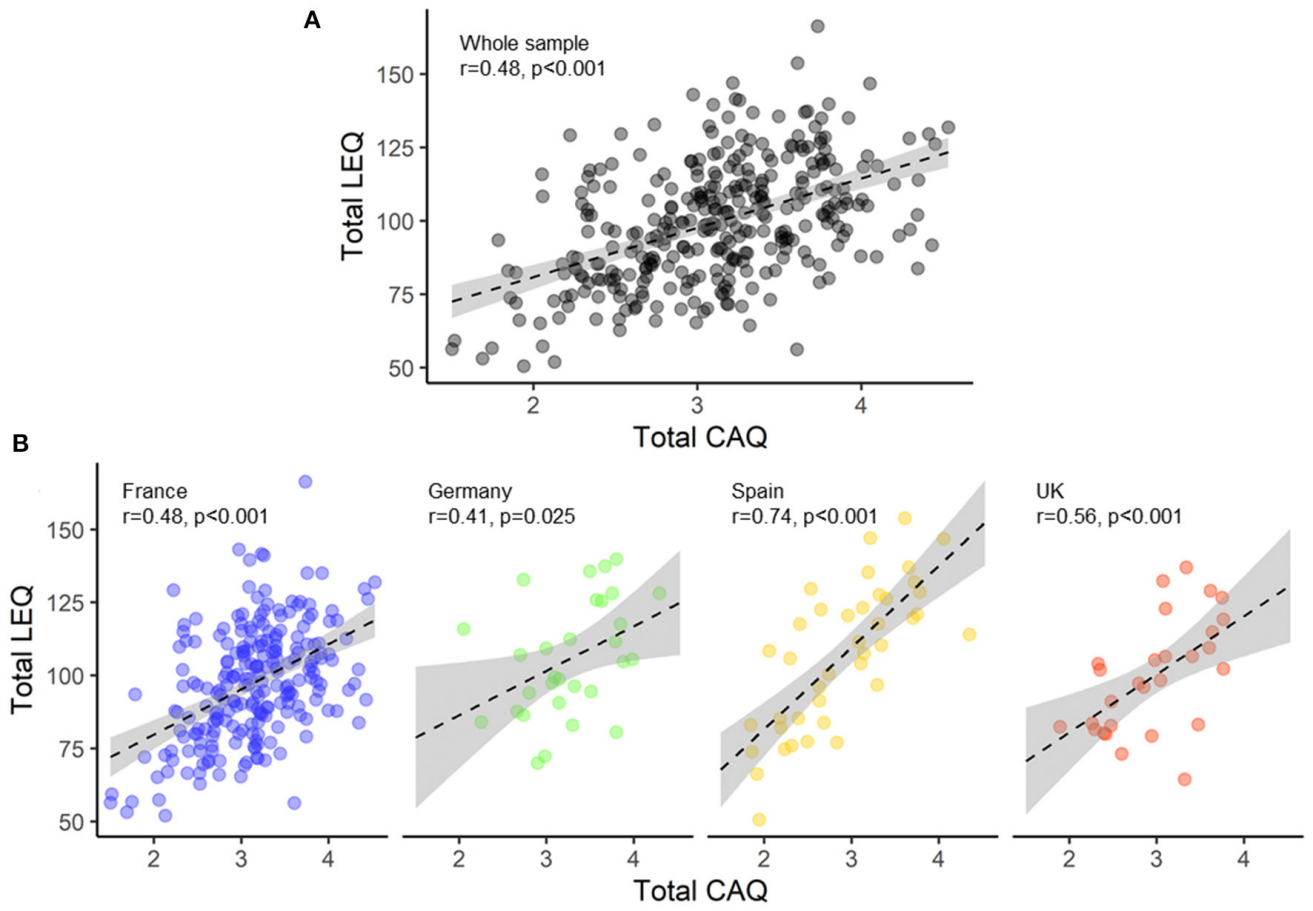
Concurrent validity: scatterplots illustrating the correlation between total LEQ and total CAQ in the whole sample (black) **(A)** and within each country (France, blue; Germany, green; Spain, yellow; United Kingdom, red) **(B)**. LEQ, Lifetime of Experiences Questionnaire; CAQ, Cognitive Activities Questionnaire; UK, United Kingdom.

Total LEQ showed moderate to strong positive associations with total CAQ (whole sample: *r* = 0.48, *p* < 0.001; France: *r* = 0.46, *p* < 0.001; Germany: *r* = 0.41, *p* = 0.025; Spain: *r* = 0.74, *p* < 0.001; UK: *r* = 0.56, *p* < 0.001) (Figure 1). The LEQ sub-scores (non-specific) also showed moderate to strong positive associations with the CAQ sub-scores within similar periods, in the whole sample and within each country (Supplementary Figure 2). However, when Bonferroni correction was applied (i.e., *p* = 0.05/9), correlations within similar periods failed to reach significance for Germany and UK.

#### Structural Validity

##### Common-Level Dimensional Structure

**Figure 2:**
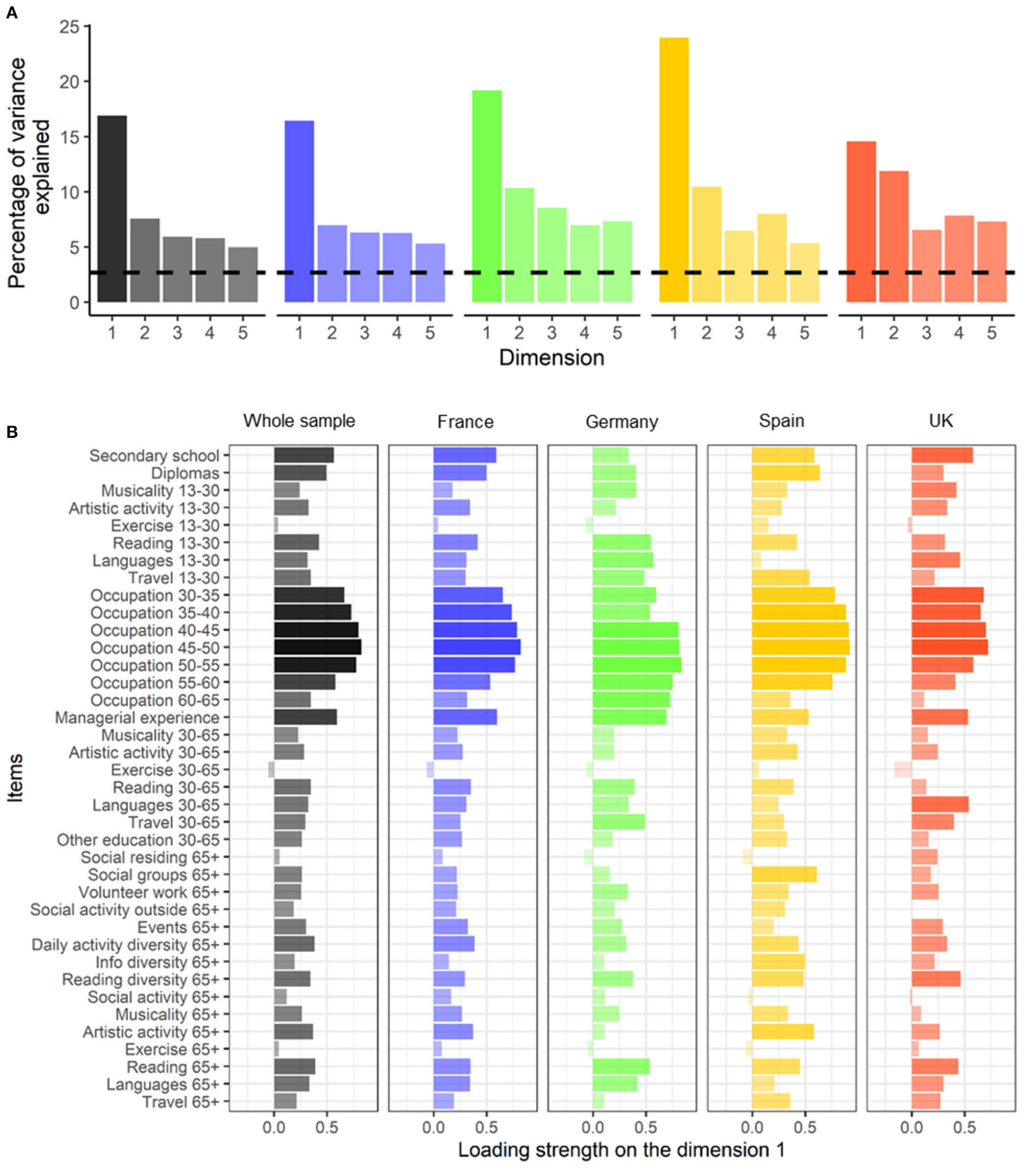
Structural validity: Scree plot of the percentage of variance explained by the first five dimensions of the LEQ **(A)** and loading strength of the LEQ items (unrotated coordinates) on the first dimension **(B)** in the whole sample (black) and within each country (France, blue; Germany, green; Spain, yellow; United Kingdom, red). Black dashed line is the common threshold eigenvalue of 1 corresponding to 2.6% variance explained (i.e., the eigenvalue divided by 38 items). 13–30, period between 13 and 30 years old; 30–65, period between 30 and 65 years old; 65+, period beyond 65 years old.

In the whole sample, we identified 12 dimensions with an eigenvalue >1 that explained 65.4% of the total variance of the LEQ. The six first dimensions explained 45.6% of the variance (Supplementary Table 4). Specifically, the first dimension explained 16.9% of the variance, followed by 7.6% for the second dimension (Figure 2A). The first dimension, which explained twice the variance of the second dimension, had strong factor loading of items related to education and occupation complexity (Figure 2B). The second dimension had strong factor loading of items related to leisure activities such as physical activity, artistic practise, reading, or practising a second language (Supplementary Figure 3).

##### Country-Level Dimensional Structure

Within each country, the 12 dimensions with eigenvalues > 1 explained 66.4, 92.6, 92.6, and 96.4% of the total variance of the LEQ for France, Germany, Spain, and the UK, respectively. The six first dimensions explained 45.8, 58.2, 60.6, and 55.6% for France, Germany, Spain, and the UK, respectively (Supplementary Table 4): the first dimension explained 16.4% (eigenvalue = 6.2), 19.2% (eigenvalue = 7.3), 24% (eigenvalue = 9.1), and 14.6% (eigenvalue = 5.5) of the variance for France, Germany, Spain, and the UK, respectively, and explained almost twice as much variance compared to the second dimension except for UK (France, 7%; Germany, 10.4%; Spain, 10.5%; UK, 11.9%) (Figure 2A). The first and the second dimensions showed similar loading patterns at the country level than at the whole sample level, suggesting that the adapted and harmonised questionnaire measures a similar construct across countries (Figure 2B).

### Association With Global Cognition and Regional Brain Volumes

**Figure 3:**
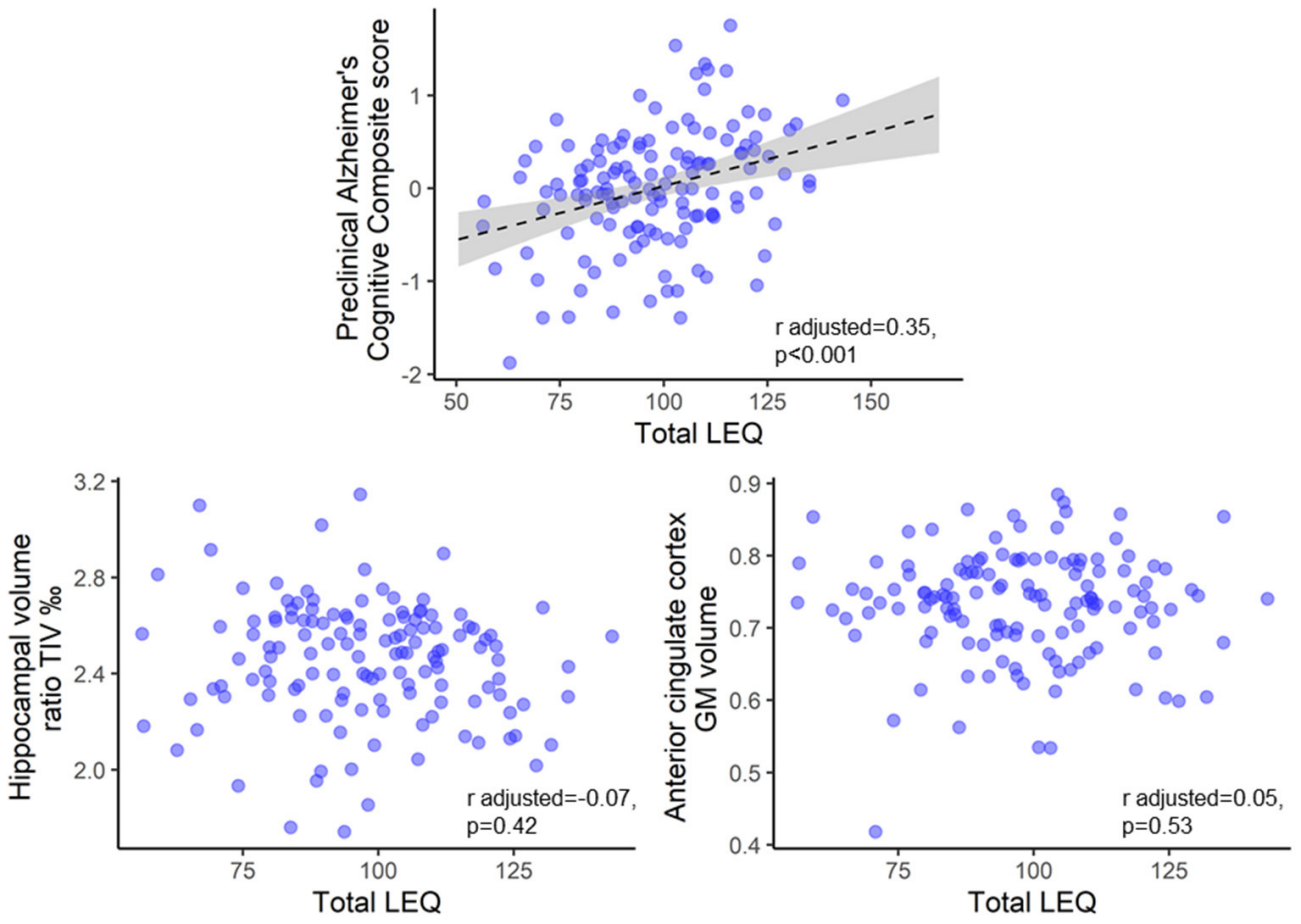
Brain and cognitive associations: scatterplot of total LEQ and the Pre-clinical Alzheimer's Cognitive Composite, the grey matter volumes of the hippocampus, and the anterior cingulate cortex at baseline. Raw data (i.e., unadjusted for age and sex) are plotted. Statistical values were adjusted for age and sex. Please note that these analyses were performed in the French Age-well sub-sample because of data availability (*n* = 135). GM, grey matter; TIV, Total Intracranial Volume.

A positive association was found between total LEQ and the PACC5 (*r* adjusted = 0.35, *p* < 0.001) (Figure 3). Additionally, positive associations were found between the sub-scores of the LEQ and the PACC5 (all *p* < 0.001) (Supplementary Figure 4).

No associations were found between total LEQ and the hippocampal volume or the anterior cingulate cortex GM volume (Figure 3). Additionally, no association was found between the sub-scores of the LEQ and the hippocampal volume or the anterior cingulate cortex GM volume (Supplementary Figure 4).

### Between-Country Differences in Complex Mental Activity Across the Life-Course

**Figure 4:**
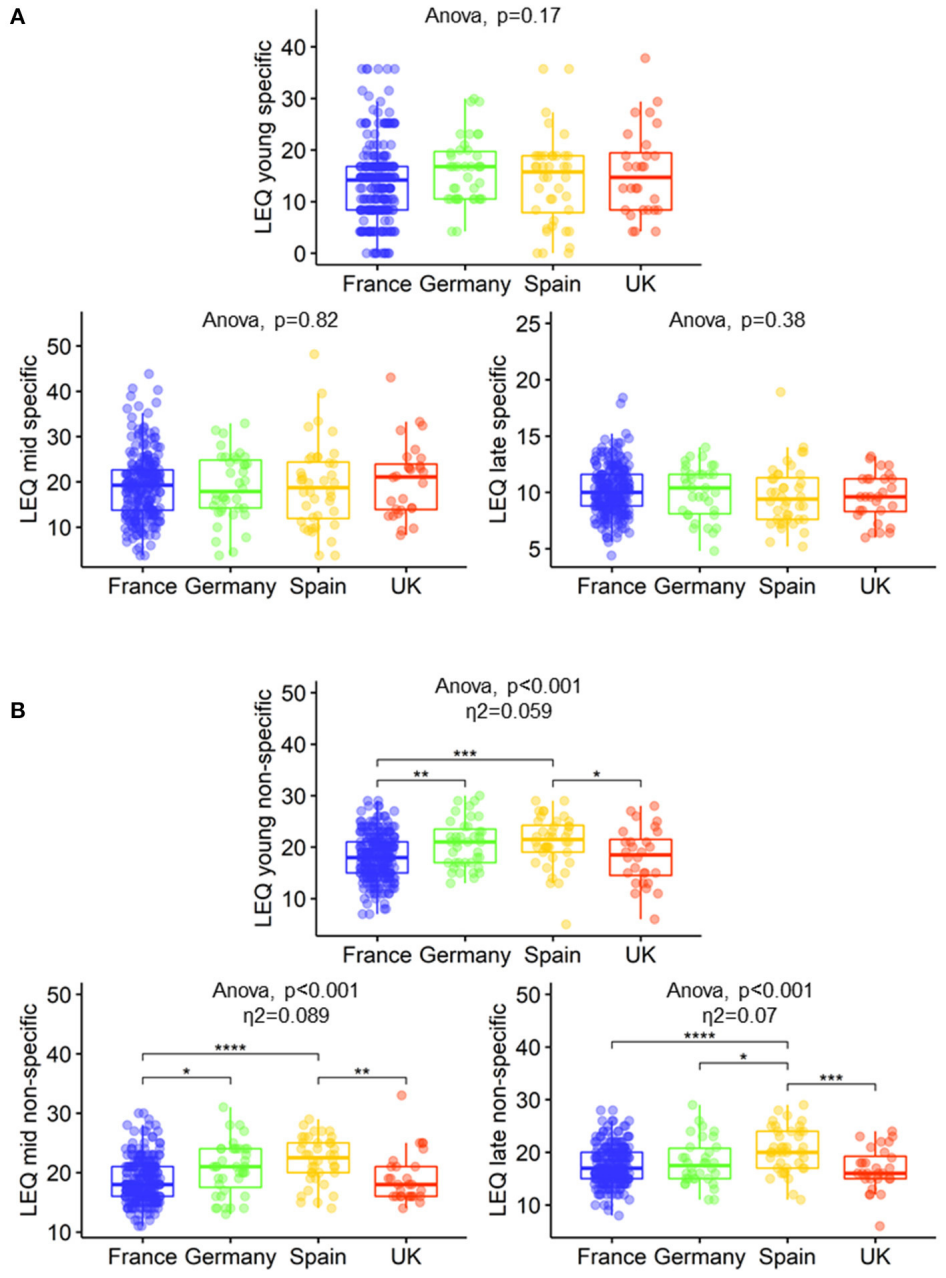
Between-country differences in education and occupation **(A)** and leisure activities engagement **(B)** of the LEQ. Boxplots show the 25th, 50th, and 75th percentiles within each country. Tukey Honestly Significant Difference (HSD) was used as a *post hoc* test: ^*^P < 0.05, ^**^P < 0.01, ^***^P < 0.001, ^****^P < 0.0001. LEQ, Lifetime of Experiences Questionnaire; UK, United Kingdom.

Between-country differences were observed for the total LEQ (*p* = 0.019, Spain > France and Germany > France), the young adulthood period (*p* = 0.01, Germany > France) and the late life period (*p* = 0.009, Spain > UK, Spain > France) (Table 1). Between-country differences were also observed for non-specific sub-scores (reflecting leisure activities engagement): young adulthood (*p* < 0.001, Spain > UK and France, Germany > France), mid-life (*p* < 0.001, Spain > UK and France, Germany > France) and late life (*p* < 0.001, Spain > UK and France and Germany) periods, but not for specific sub-scores (reflecting education and occupation) (Figure 4).

Similar to what was observed for the LEQ, between-country differences were observed on the CAQ in the early period between Germany and France (Germany > France). Additional differences were observed in late life (but not mid-life) using the CAQ sub-scores as shown in Supplementary Figure 5.

## Discussion

In this cross-sectional study, we assessed the validity of an adapted and harmonised version of the LEQ to assess differences in complex mental activity across the life-course in four European countries. Our main results demonstrated that the adapted LEQ questionnaire shows (i) concurrent validity evidenced by moderate to strong linear correlations with the CAQ and a multidimensional structure consistent with that observed in the original LEQ and consistent across countries, (ii) associations with global cognition but not with brain volumetric measures, and (iii) between-country differences in life-course mental activity, specifically, in the engagement in leisure activities.

Our results show positive associations between total LEQ and total CAQ in the whole sample and within countries. Positive associations were also found between similar sub-scores of the LEQ and the CAQ measuring complex mental activity and cognitively stimulating activities, respectively, in relatively similar life periods. The CAQ is a well-known and validated tool in the dementia field that measures cognitively stimulating activities across the life-course (Wilson et al., 2003). In contrast to the LEQ, the CAQ was developed to enhance the applicability of the scale among persons with diverse cultural and socioeconomic background. Our findings thus demonstrate that the adapted LEQ shows good concurrent validity in the whole sample–which is in line with the previous validation study (Valenzuela and Sachdev, 2007)–and within each country, supporting a cross-cultural equivalence of the measurement. Although, the two tests showed a good linear correlation overall, there were some weak associations. These weak associations between the LEQ and the CAQ within similar periods (e.g., Germany and UK) could be explained by reduced statistical power due to smaller sample size in some countries, limited items in each CAQ period, slight differences in the content of the questions (e.g., physical activity is not assessed in the CAQ) and differences in the time periods assessed (i.e., questions about a specific age in the CAQ, e.g., age 18 vs. questions covering a full period in the LEQ, e.g., from 13 to 30).

The dual multiple factor analysis revealed a multidimensional structural pattern of the adapted LEQ in the whole sample, which is consistent with the original paper by Valenzuela and Sachdev (2007) and the multidimensionality of the determinants of CR. The multidimensional structural pattern was consistent across countries, supporting the cross-cultural equivalence of the structure of the adapted questionnaire. In the adapted LEQ, the first dimension accounted for almost twice the variance that the second dimension explained, and mainly loaded on education and occupation items while the second dimension loaded on leisure activities (e.g., artistic practise and reading). These findings are in line with the previous study by Valenzuela and Sachdev (2007) showing (i) a similar variance explained for the first dimension (14%) and for dimensions with eigenvalue > 1 (81%) and (ii) a similar multidimensional structure of the LEQ (>10 dimensions), which overall suggests that it is highly reproducible. Furthermore, the first and the second dimension shared similar patterns of loaded items within countries, which demonstrates a cross-cultural equivalence of the structure of the adapted LEQ. Taken together, these results are in line with previous evidence, strengthen the original structural validity with a larger sample, and present new evidence showing the stability of the multidimensional construct across countries.

Interestingly, greater total LEQ score and sub-scores were associated with better Pre-clinical Alzheimer's Cognitive Composite score, a measure of global cognition. Previous studies have shown that healthy older adults with greater LEQ scores showed better global cognition (Paplikar et al., 2020) and less cognitive decline over time (Valenzuela and Sachdev, 2007), suggesting a protective role of complex mental activity across the life-course. Although longitudinal cognitive decline was not assessed here, these findings add to the evidence showing that complex mental activity across the life-course contribute to better global cognition in older adults. In contrast, we did not find associations between total LEQ and hippocampal or anterior cingulate cortex volume. Although these AD-sensitive regions are known to be associated with CR proxies (Arenaza-Urquijo et al., 2013), a recent study did not find a relationship (Kalzendorf et al., 2020). Our findings with brain measurements may be explained by the cross-sectional nature of the study and the cognitive status of the participants. Complex mental activity or managerial experience measured by the LEQ were previously associated with rate of hippocampal atrophy in healthy older adults (Valenzuela et al., 2008; Suo et al., 2012). However, only managerial experience, but not complex mental activity (total LEQ), was cross-sectionally associated with hippocampal volume in healthy older adults (Valenzuela et al., 2008; Suo et al., 2012;) and patients with non-amnestic mild cognitive impairment (Suo et al., 2017). Overall, our results suggest that life-course complex mental activity contribute to better global cognition. However, even if we did not find associations with brain measurements, the literature suggests that life-course complex mental activity may act as a protective factor against cognitive decline and brain atrophy, and particularly hippocampal atrophy. These relationships results may only be measurable longitudinally or in cognitively impaired older adults. It is also possible that complex mental activities show regional effects on specific sub-regions of the hippocampus and cingulate cortex, or on brain functional measurements (e.g., glucose metabolism or functional connectivity).

Between-country differences were observed for total LEQ and sub-scores suggesting that the adapted LEQ is able to capture differences across life-course and across countries. Interestingly, these differences were specific to leisure activities engagement rather than to education and occupation, in fact, besides the LEQ, there was no between-country difference in education. Similar results to those found with the LEQ (differences in leisure activities engagement) were also observed with the CAQ–a questionnaire that does not include education and occupation assessments (Wilson et al., 2003). While between-country differences in education and socio-economic status can contribute to differences in AD prevalence/incidence (Prince et al., 2013; Rizzi et al., 2014), other modifiable risk factors such as social interaction or physical activity–measured in the LEQ non-specific sub-scores–could also contribute to AD prevalence/incidence (Livingston et al., 2020). Education, socio-economic status, and occupation are systematically evaluated in observational and interventional studies. However, the LEQ captures a range of additional factors which may also contribute to AD prevalence/incidence. Cognitive reserve is nurtured by a variety of exposures across the life-course (Scarmeas and Stern, 2003) which may influence the outcomes of the studies including cognition; ageing and AD biomarkers; or AD incidence/prevalence estimates (Menardi et al., 2018; Nelson et al., 2021). The LEQ seems therefore fundamental to pinpoint the multidimensional aspects of CR as well as the variety of exposures that may increase it. To our knowledge, no previous study has investigated between-country differences in complex mental activity. Although our findings should be interpreted with caution given the different size between samples and recruitment strategies (from the community or from medical facilities), we were able to detect differences in cognitively normal older adults over 60 years old. Further studies investigating between-country comparisons in lifestyle, and particularly socio-behavioural proxies of CR, would be helpful to investigate whether the exposures that increase reserve, as well as the CR mechanisms, are different across cultures and countries. This could inform country-specific interventions and policies to promote healthy ageing.

The main strength of this study is the relatively large and international sample included (four different countries), including participants with available clinical, cognitive, and neuroimaging data. With this study, we have been able to harmonise and adapt the LEQ across countries and show good concurrent and structural validity, thus strengthening previous evidence (Valenzuela and Sachdev, 2007). This cross-cultural adaptation and validation of the LEQ is particularly timely, given recent calls for improvements to CR measures (Kartschmit et al., 2019; Landenberger et al., 2019). We believe that this effort will aid the comparison of complex mental activity across countries in international studies.

This study is not free of limitations. Although the factor analysis demonstrated similar structures across countries, some items showed no variability in some countries. Further, the UK sample was smaller than the samples from the other countries. The interventional design of the Medit-ageing project prevented us from using longitudinal data to assess content validity (CR mechanism) and reliability (test-retest) of the questionnaire. Due to the study design and recruitment strategies, the results of this paper cannot be generalised to the wider population. The scope of this adapted and validated version is limited to the four countries included here. Although adapting the LEQ for European countries with a similar cultures was relatively straightforward, adaptations for countries with rather different cultures (e.g., Non-European countries) may prove more challenging (Paplikar et al., 2020).

## Conclusion

Our results demonstrated that the adapted LEQ questionnaire for France, Germany, Spain, and the UK, shows (i) concurrent validity–evidenced by moderate to strong linear correlations with the CAQ and a multidimensional structure, consistent with the original LEQ and across countries, (ii) associations with global cognition but not with brain measures, and (iii) between-country differences in life-course mental activity, specifically in the engagement in leisure activities. The LEQ is a promising tool to assess the multidimensional construct of CR. These adapted versions of the LEQ can be used as a measure of socio behavioural determinants of CR in older adults across countries. Further longitudinal studies are warranted to test its clinical utility.

## Data Availability Statement

The datasets presented in this article are not readily available because IMAP data are made available upon request to the sponsor (Caen University Hospital) and the principal investigator (chetelat@cyceron.fr), and MEDIT-Ageing/Silver Sante Study data (SCD-well and Age-well trials) are made available upon request following a formal data sharing agreement and approval by the consortium and executive committee (https://silversantestudy.eu/). Requests to access the datasets should be directed to chetelat@cyceron.fr or https://silversantestudy.eu/.

## Ethics Statement

The studies involving human participants were reviewed and approved by Ethics Committees and regulatory agencies of all centres: Caen, France (Comité de Protection des Personnes CPP Nord-Ouest III, Caen; trials registration numbers: EudraCT: 2016-002441-36 and 2011-A01493-38; IDRCB: 2016-A01767-44 and 2011-A01493-38; ClinicalTrials.gov Identifiers: NCT02977819 and NCT01638949); London, UK (Queen Square Research Ethics Committee: No 17/LO/0056 and Health Research Authority National Health Service, IRAS project ID: 213008); Lyon, France (Comité de Protection des Personnes Sud-Est II Groupement Hospitalier Est: No. 2016-30-1 and Agence Nationale de Sécurité du Médicament et des Produits de Santé: IDRCB 2016-A01298-43); Cologne, Germany (Ethikkommission der Medizinischen Fakultät der Universität zu Köln: No. 17-059); and Barcelona, Spain (Comité Etico de Investigacion Clinica del Hospital Clinic de Barcelona: No. HCB/2017/0062). The patients/participants provided their written informed consent to participate in this study.

## Author Contributions

VO, NM, GC, and EA-U contributed to conception and design of the study. VO and EA-U wrote the first draft manuscript and performed the statistical analysis. VO, NM, A-KS, NC-P, OK, JG, RF, MV, MW, and EA-U contributed to administrative, technical, and material support. All authors took public responsibility for the whole or part of the content, contributed to the acquisition, analysis and interpretation of data, manuscript revision, and read and approved the submitted version.

## Funding

This study was supported by the European Union's Horizon 2020 Research and Innovation Program (Grant agreement no. 667696), Fondation Plan Alzheimer (Alzheimer Plan 2008–2012), Programme Hospitalier de Recherche Clinique (PHRCN 2011-A01493-38 and PHRCN 2012 12-006-0347), Agence Nationale de la Recherche (LONGVIE 2007), Institut National de la Santé et de la Recherche Médicale (INSERM), Région Normandie, Association France Alzheimer, Fondation Alzheimer, Fondation Recherche Alzheimer, Fondation d'Entreprise MMA des Entrepreneurs du Futur, and Fondation Entrepreneurs MMA. EA-U holds a Ramón y Cajal fellowship (RYC2018-026053-I). The funders and sponsor had no role in the design and conduct of the study, collection, management, analysis, and interpretation of the data, preparation, review, or approval of the manuscript, and decision to submit the manuscript for publication.

## Conflict of Interest

MV was employed by company Skin2Neuron Pty Ltd. The remaining authors declare that the research was conducted in the absence of any commercial or financial relationships that could be construed as a potential conflict of interest.

## Publisher's Note

All claims expressed in this article are solely those of the authors and do not necessarily represent those of their affiliated organizations, or those of the publisher, the editors and the reviewers. Any product that may be evaluated in this article, or claim that may be made by its manufacturer, is not guaranteed or endorsed by the publisher.
