# Supplementary material for: Harmonisation and Between-Country Differences of the Lifetime of Experiences Questionnaire in Older Adults
Source: Front Aging Neurosci. 2021 Oct 14;13:740005. doi: 10.3389/fnagi.2021.740005 (PMC8551756; doi:10.3389/fnagi.2021.740005)
Supplement: Supplementary file 1 [file Data_Sheet_1.PDF]

Supplementary Material

Supplementary Figures

Figure S1. Illustration of measurements included in the LEQ and the time frame (A) and the flow chart of the analysis (B)

Figure S2. *Concurrent validity*: Correlations between the LEQ (non-specific) and the CAQ sub-scores in the whole sample and within each country

Figure S3. *Structural validity*: Loading strength of the LEQ items (unrotated coordinates) on the second dimension

Figure S4. *Brain and cognitive associations*: scatterplot of the sub-scores (early, mid and late) of the LEQ and the Preclinical Alzheimer's Cognitive Composite, the grey matter volumes of the hippocampus and the anterior cingulate cortex at baseline

Figure S5. Boxplots illustrating between-country differences in lifespan cognitive activities of the CAQ (Early, Mid, Late)

Supplementary Tables

Table S1: Secondary years of school and associated scoring across Australia, France, Germany, Spain and the United-Kingdom

Table S2: The Australian Standard Classification of Occupations (ASCO) and the International Standard Classification of Occupations (ISCO), comparison and associated

Table S3: Illustration allowing the comparison between primary and secondary education across Australia (original LEQ), France, Spain, the United-Kingdom, and East-West Germany for older adults that are currently >60 yrs

Table S4: Eigenvalues and variances of the 12 dimensions of the dual multiple factor analysis of the LEQ

Table S5: Post-secondary levels, diplomas and associated scoring across Australia, France, Germany, Spain and the United-Kingdom.

Supplementary figures

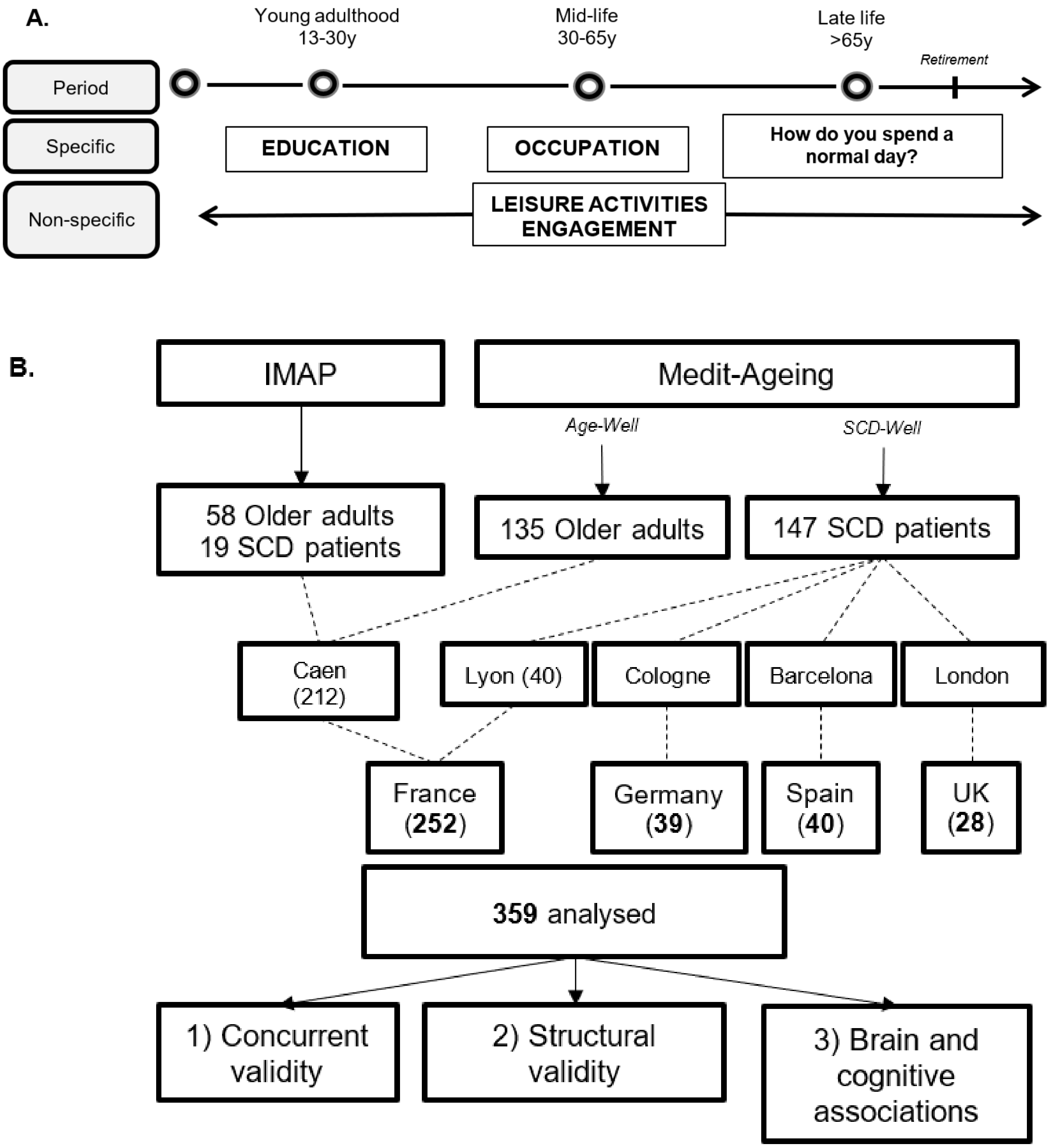

Figure S1. Illustration of measurements included in the LEQ and the time frame **(A)** and the flow chart of the analysis **(B)**. Abbreviations: LEQ, Lifetime of Experiences Questionnaire; SCD, Subjective Cognitive Decline; UK, United-Kingdom.

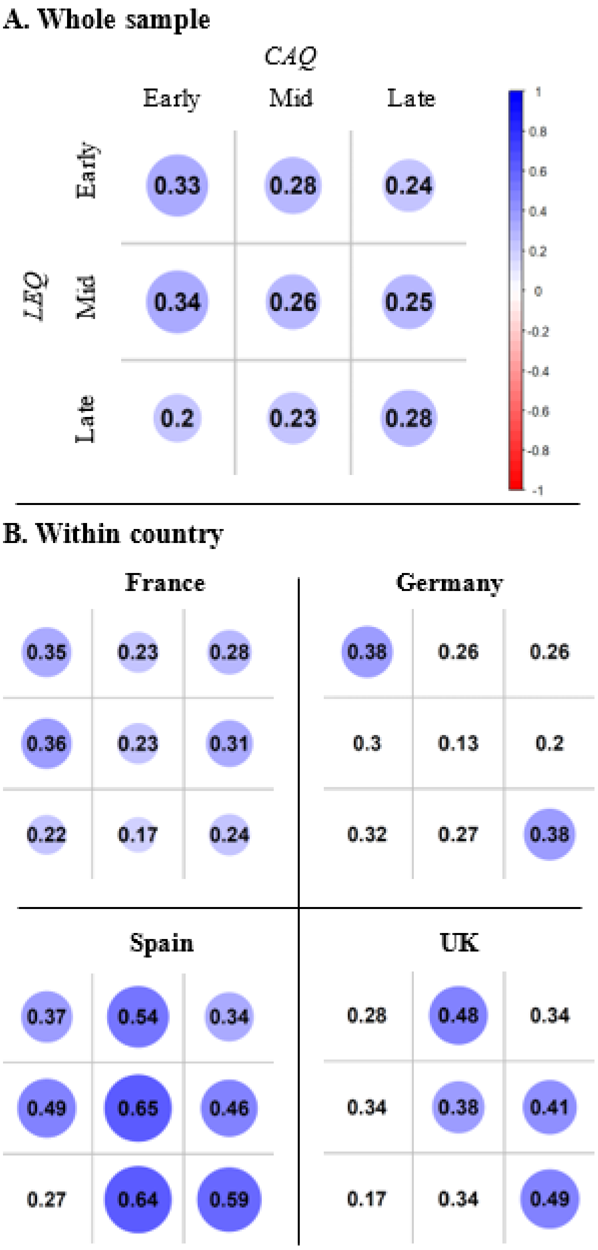

Figure S2. *Concurrent validity*: Correlations between the LEQ (non-specific) and the CAQ sub-scores in the whole sample **(A)** and within each country **(B)**. Significant positive Pearson's coefficients are displayed with blue circle (p value <0.05). Abbreviations: LEQ, Lifetime of Experience Questionnaire; CAQ, Cognitive Activities Questionnaire; UK, United-Kingdom.

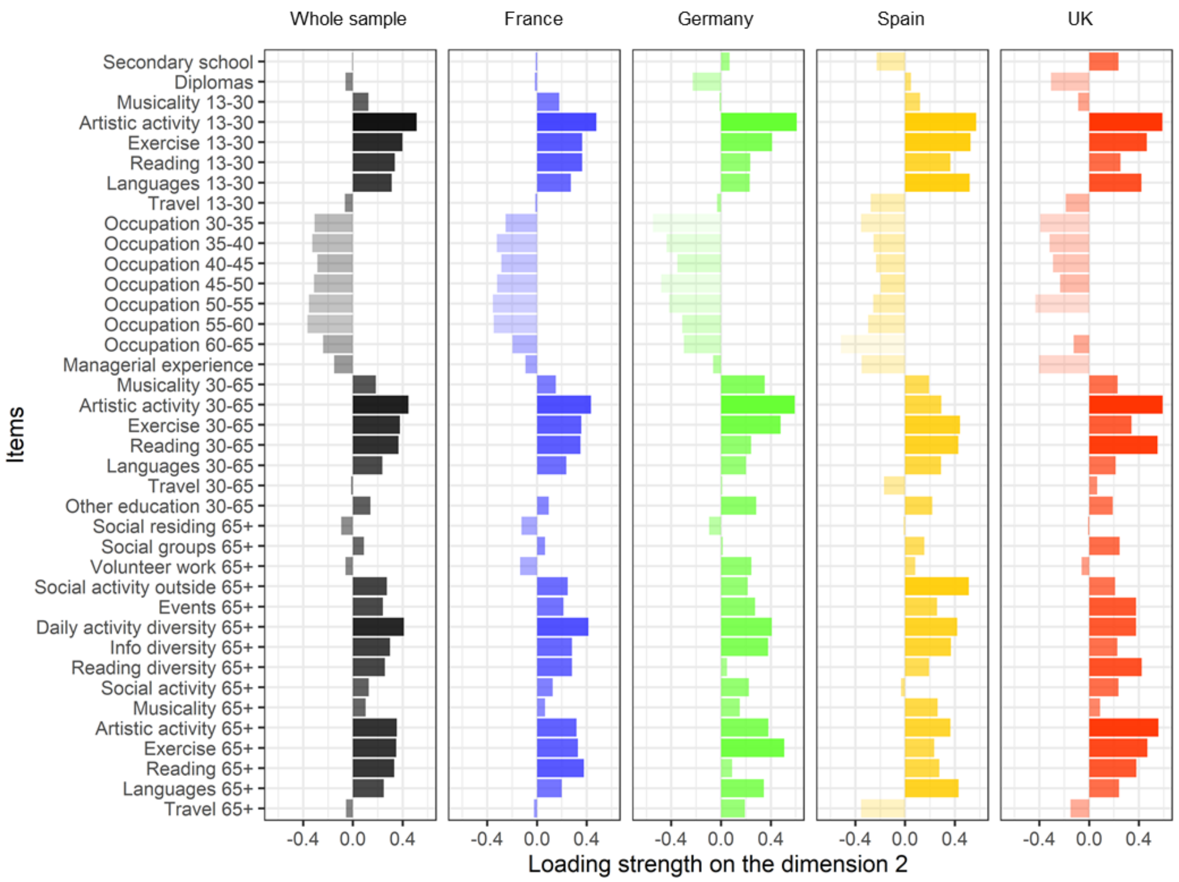

Figure S3. *Structural validity*: Loading strength of the LEQ items (unrotated coordinates) on the second dimension in the whole sample (black) and within each country (France, blue; Germany, green; Spain, yellow; United Kingdom (UK), red). Abbreviations: 13-30, period between 13 and 30 years old; 30-65, period between 30 and 65 years old; 65+, period beyond 65 years old.

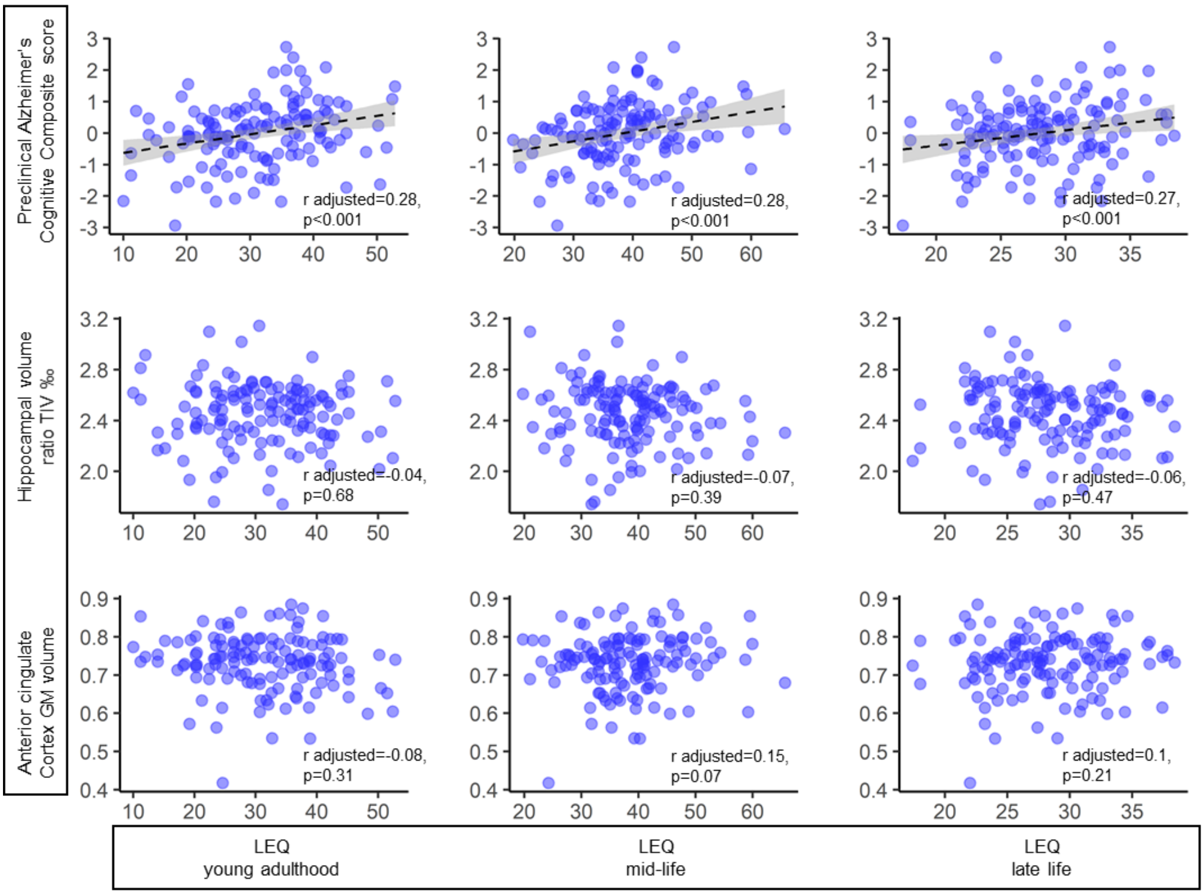

Figure S4. *Brain and cognitive associations*: scatterplot of the sub-scores (early, mid and late) of the LEQ and the Preclinical Alzheimer's Cognitive Composite, the grey matter volumes of the hippocampus and the anterior cingulate cortex at baseline. Raw data (i.e., unadjusted for age and sex) are plotted. Statistical values were adjusted for age and sex. Please note that these analyses were performed in the French Age-well sub-sample because of data availability (n=135). Abbreviations: GM, grey matter; TIV, Total Intracranial Volume.

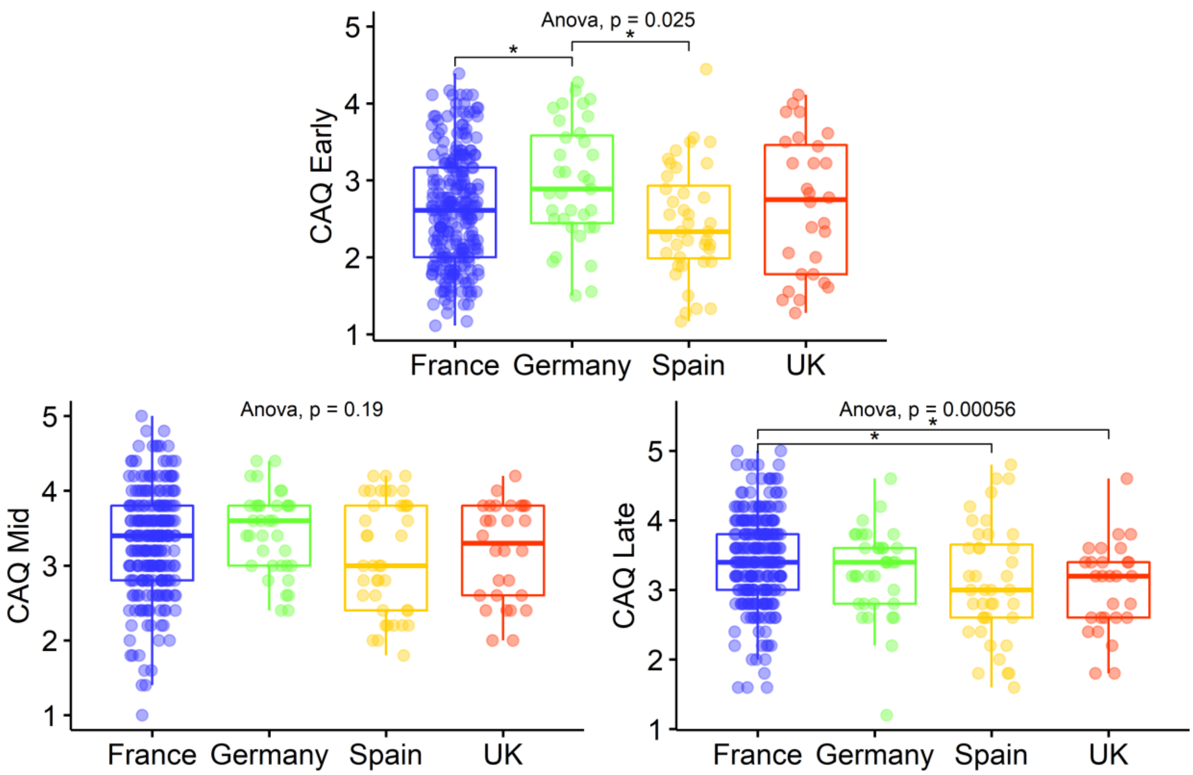

Figure S5. Between-country differences in lifespan cognitive activities of the CAQ (Early, Mid, Late). The boxplots show 25th, 50th and 75th percentiles within each country. Tukey Honestly Significant Difference (HSD) was used as a post hoc test: *P < 0.05, **P < 0.01, *** P < 0.001, ****P < 0.0001. Abbreviations: CAQ, Cognitive Activities Questionnaire; UK, United-Kingdom.

Supplementary tables

Table S1: Secondary school duration (in years) and associated scoring across Australia, France, Germany, Spain and United-Kingdom.

	Australia (original)	France	Germany[a]	Spain	United Kingdom
Score	Secondary school yrs	Secondary school yrs	Primary + secondary school yrs	Secondary school yrs	Secondary school yrs
+ 0	0, 1	0, 1, 2	6, 7	0, 1	0, 1
+ 4	2, 3, 4	3, 4, 5	8, 9, 10	2, 3, 4	2, 3, 4
+ 8	5, 6	6, 7	11, 12	5, 6	5, 6

Note: [a]because of differences between East and West Germany, primary education is used in addition to secondary school to score. The last 2 years of secondary school have the maximum score (+8).

Table S2*:* The Australian Standard Classification of Occupations (ASCO) and the International Standard Classification of Occupations (ISCO), comparison and associated scoring. Differences are represented in grey colour.

ASCO Classification	ASCO Original Scoring	ISCO Classification	ISCO Scoring
1. Managers and administrators	+ 10	1. Managers	+ 10
2. Professionals	+ 9	2. Professionals	+ 9
3. Associate professionals	+ 8	3. Technicians and Associate Professionals	+ 8
4. Tradespersons and related workers	+ 7	7. Craft and Related Trades Workers	+ 4
5. Advanced Clerical and service workers	+ 6	4. Clerical Support Workers	+ 7
6. Intermediate clerical, sales, and service workers	+ 5	5. Services and Sales Workers	+ 6
7. Intermediate production and transport workers	+ 4	8. Plant and Machine Operators and Assemblers	+ 3
8. Elementary clerical, sales, and service workers	+ 3	9. Elementary Occupations	+ 2
9. Labourers and related workers	+ 2	6. Skilled Agricultural, Forestry and Fishery Workers	+ 5
10. None/stay-at- home parent	+ 1	10. None/stay-at- home parent	+ 1
11. Armed Forces Occupations	+ 1	11. Armed Forces Occupations	10, 9, 8 (depending of the grade)

Table S3: Illustration allowing the comparison between primary and secondary education across Australia (original LEQ), France, Spain, United-Kingdom, and East-West Germany for older adults that are currently >60 yrs.

Grade	0	1	2	3	4	5	6	7	8	9	10	11	12	
Age	5-6	6-7	7-8	8-9	9-10	10-11	11-12	12-13	13-14	14-15	15-16	16-17	17-18	
Australia		Primary school						Secondary school						Certificate
France		Primary school					Secondary school							Certificate
East Germany[a]		Primary school								Secondary school				Certificate
West Germany[a]		Primary school				Secondary school						Secondary school 2nd phase		Certificate
Spain[b]		Primary school				Secondary school				Secondary school 2nd phase (option)		Certificate		
United-Kingdom[c]		Primary school						Secondary school						Certificate

Note: Education system changed in 1990[a], 1953[b], 1944[c].

Table S4: Eigenvalues and variances of the 12 dimensions of the dual multiple factor analysis of the LEQ.

	Whole sample			France			Germany			Spain			United-Kingdom		
Dim	Eig	% var	Cum.	Eig	% var	Cum.	Eig	% var	Cum.	Eig	% var	Cum.	Eig	% var	Cum.
1	6.4	16.9	16.9	6.2	16.4	16.4	7.3	19.2	19.2	9.1	24.0	24.0	5.5	14.6	14.6
2	2.9	7.6	24.5	2.7	7.0	23.4	3.9	10.4	29.5	4.0	10.5	34.4	4.5	11.9	26.4
3	2.3	5.9	30.4	2.4	6.3	29.7	3.2	8.5	38.1	2.5	6.5	40.9	2.5	6.6	33.0
4	2.2	5.8	36.2	2.4	6.3	36.0	2.6	7.0	45.0	3.0	8.0	48.9	3.0	7.8	40.9
5	1.9	5.0	41.2	2.0	5.3	41.3	2.8	7.3	52.4	2.0	5.3	54.2	2.8	7.3	48.2
6	1.7	4.4	45.6	1.7	4.5	45.8	2.2	5.8	58.2	2.4	6.3	60.6	2.8	7.5	55.6
7	1.5	3.8	49.4	1.5	4.1	49.9	2.3	5.9	64.1	1.6	4.3	64.9	2.8	7.3	63.0
8	1.4	3.6	53.0	1.3	3.5	53.3	2.6	6.8	70.8	1.7	4.5	69.4	2.7	7.2	70.2
9	1.3	3.3	56.3	1.3	3.4	56.8	2.1	5.4	76.2	2.9	7.5	77.0	2.3	6.1	76.3
10	1.2	3.2	59.5	1.3	3.5	60.2	2.2	5.7	81.9	1.9	5.1	82.0	1.9	5.0	81.3
11	1.2	3.1	62.5	1.1	3.0	63.2	1.9	5.0	86.9	2.2	5.8	87.9	2.7	7.1	88.4
12	1.1	2.9	65.4	1.2	3.1	66.4	2.2	5.7	92.6	1.8	4.7	92.6	3.1	8.0	96.4

Note: Eigenvalues < 1 were not reported. Abbreviations: LEQ, Lifetime of Experience Questionnaire; Dim, Dimension; Eig, Eigenvalue; % var, percentage of variance explained; Cum. % var, cumulative percentage of variance explained.

Table S5: Post-secondary levels, diplomas and associated scoring across Australia, France, Germany, Spain and United-Kingdom. Score do not change compared to the Australian version

Scoring	Australia	France	Germany	Spain	United-Kingdom
+ 4	Clerical, admin training (0-1y)	Emploi de bureau administratif (0-1y)	Büro-, Verwaltungs- oder Buchführungsausbildung (0-1y)	Administrativo (0-1y)	Clerical, admin training (0-1y)
+ 6	Clerical, admin training (1-2y)	Emploi de bureau administratif (1-2y)	Büro-, Verwaltungs- oder Buchführungsausbildung (1-2y)	Administrativo (1-2y)	Clerical, admin training (1-2y)
+ 4	Business course (0-1y)	Métier de la gestion et du commerce (0-1y)	Geschäftsführungskurs / Gewerblich / technische Berufsausbildung (0-1y)	Cursos de empresa (0-1y)	Business course (0-1y)
+ 6	Business course (1-2y)	Métier de la gestion et du commerce (1-2y)	Geschäftsführungskurs / Gewerblich / technische Berufsausbildung (1-2y)	Cursos de empresa (1-2y)	Business course (1-2y)
+ 8	Trade apprenticeship	Apprentissage	Gewerbliche Ausbildung / Sonstige Berufsausbildung	Aprendizaje comercial	Trade apprenticeship
+ 6	Other technical, business course (1-2y)	Autres formations techniques et diplomantes (1-2y)	Soziale Berufsausbildung / Sonstiger weiterführender Studiengang (1-2y)	Otros cursos tecnicos (1-2y)	Other technical, business course (1-2y)

+ 8	Other technical, business course (3-4y)	Autres formations techniques et diplomantes (3-4y)	Sonstiger weiterführender Studiengang (3-4y)	Otros cursos tecnicos (3-4y)	Other technical, business course (3-4y)
+ 8	College Diploma	Licence, DEUG	Hochschuldiplom / Bachelor	Estudios Universitarios	College Diploma
+ 10	University Undergraduate (3 or 4 years degree)		Universitätsstudium (3- oder 4-jähriges Studium)		University Undergraduate (3 or 4 years degree)
+ 10	University Undergraduate (5 or 6 years degree e.g. Medecine)		Universitätsstudium (5- oder 6-jähriges Studium, z.B. Medizin)		University Undergraduate (5 or 6 years degree e.g. Medicine)
+ 8	University Masters	Masters, maitrise, DEA	Universitärer Master-Abschluss	Masterias	University Masters
+ 10	University PhD, Doctorate	Doctorat	Doktorgrad einer Universität/Promotion	Doctorado	University PhD, Doctorate
+ 0	None	Aucun	Nicht	No	None
