# Supplementary material for: Harmonisation and Between-Country Differences of the Lifetime of Experiences Questionnaire in Older Adults
Source: Front Aging Neurosci. 2021 Oct 14;13:740005. doi: 10.3389/fnagi.2021.740005 (PMC8551756; doi:10.3389/fnagi.2021.740005)
Supplement: Supplementary file 2 [file Table_1.DOCX]

ª**List of all members of the Medit-Ageing Research Group**

| First Name | Middle Initial | Last Name | Highest Degree | Institution | Location | Email |
| --- | --- | --- | --- | --- | --- | --- |
| Eider | M. | ARENAZA-URQUIJO | PhD | INSERM  Barcelonabeta Brain Research Center | France  Spain | [eiderarenaza@gmail.com](mailto:eiderarenaza@gmail.com) |
| Florence |  | ALLAIS | BA | INSERM | France | [florence.allais@u-bordeaux.fr](mailto:florence.allais@u-bordeaux.fr) |
| Claire |  | ANDRÉ | PhD | INSERM | France | [andre@cyceron.fr](mailto:andre@cyceron.fr) |
| Julien |  | ASSELINEAU | PhD | INSERM | France | [julien.asselineau@u-bordeaux.fr](mailto:julien.asselineau@u-bordeaux.fr) |
| Romain |  | BACHELET | PhD | HCL | France | [romain.bachelet@chu-lyon.fr](mailto:romain.bachelet@chu-lyon.fr) |
| Sebastian |  | BAEZ LUGO | MSc | UNIGE | Switzerland | [sebastian.baez@etu.unige.ch](mailto:sebastian.baez@etu.unige.ch) |
| Thorsten |  | BARNHOFER | PhD | EXETER | United-Kingdom | thorsten.barnhofer@me.com |
| Martine |  | BATCHELOR | - | independent | France | martine.batchelor@club-internet.fr |
| Axel |  | BEAUGONIN | - | independent | France | [axel.beaugonin@gmail.com](mailto:axel.beaugonin@gmail.com) |
| Alexandre |  | BEJANIN | PhD | INSERM | France | [bejanin@cyceron.fr](mailto:bejanin@cyceron.fr) |
| Maëlle |  | BOTTON | MSc | INSERM | France | [botton@cyceron.fr](mailto:botton@cyceron.fr) |
| Pierre |  | CHAMPETIER | MSc | UNICAEN | France | [champetier@cyceron.fr](mailto:champetier@cyceron.fr) |
| Gaël |  | CHÉTELAT | PhD | INSERM | France | [chetelat@cyceron.fr](mailto:chetelat@cyceron.fr) |
| Anne |  | CHOCAT | MD | INSERM | France | [annechocat@orange.fr](mailto:annechocat@orange.fr) |
| Nina |  | COLL-PADROS | MSc | IDIBAPS | Spain | [NICOLL@clinic.ub.es](mailto:NICOLL@clinic.ub.es) |
| Fabienne |  | COLLETTE | PhD | ULG | Belgium | [f.collette@uliege.be](mailto:f.collette@uliege.be) |
| Sophie |  | DAUTRICOURT | MD, MSc | UNICAEN | France | [dautricourt@cyceron.fr](mailto:dautricourt@cyceron.fr) |
| Robin |  | DE FLORES | PhD | INSERM | France | [deflores@cyceron.fr](mailto:deflores@cyceron.fr) |
| Vincent |  | DE LA SAYETTE | MD, PhD | CHU Caen | France | [delasayette-v@chu-caen.fr](mailto:delasayette-v@chu-caen.fr) |
| Pascal |  | DELAMILLIEURE | MD, PhD | CHU Caen | France | [delamillieure-p@chu-caen.fr](mailto:delamillieure-p@chu-caen.fr) |
| Marion |  | DELARUE | MSc | INSERM | France | [delarue@cyceron.fr](mailto:delarue@cyceron.fr) |
| Harriet |  | DEMNITZ-KING | MSc | UCL | United-Kingdom | [h.demnitz-king@ucl.ac.uk](mailto:h.demnitz-king@ucl.ac.uk) |
| Stéphanie |  | EGRET | MSc | INSERM | France | [egret@cyceron.fr](mailto:egret@cyceron.fr) |
| Francesca |  | FELISATTI | MSc | INSERM | France | [felisatti@cyceron.fr](mailto:felisatti@cyceron.fr) |
| Eglantine |  | FERRAND DEVOUGE | MD, MSc | INSERM | France | [eglantine.ferrand-devouge@univ-rouen.fr](mailto:eglantine.ferrand-devouge@univ-rouen.fr) |
| Eric |  | FRISON | MD, PhD | INSERM | France | [Eric.Frison@u-bordeaux.fr](mailto:Eric.Frison@u-bordeaux.fr) |
| Francis |  | GHEYSEN | MD | independent | France | [Fgheysen@aol.com](mailto:Fgheysen@aol.com) |
| Karine |  | GOLDET | PhD | HCL | France | [karine.goldet@chu-lyon.fr](mailto:karine.goldet@chu-lyon.fr) |
| Julie |  | GONNEAUD | PhD | INSERM | France | [gonneaud@cyceron.fr](mailto:gonneaud@cyceron.fr) |
| Deborah |  | HORNEY | MSc | UCL | United-Kingdom | [d.horney@ucl.ac.uk](mailto:d.horney@ucl.ac.uk) |
| Thien (Titi) |  | HUONG TRAN (DOLMA) | - | INSERM | France | [titidolma1@gmail.com](mailto:titidolma1@gmail.com) |
| Frank |  | JESSEN | MD, PhD | UKK | Germany | [frank.jessen@uk-koeln.de](mailto:frank.jessen@uk-koeln.de) |
| Agathe |  | JORET-PHILIPPE | MSc | INSERM | France | [joret@cyceron.fr](mailto:joret@cyceron.fr) |
| Olga |  | KLIMECKI | PhD | UNIGE | Switzerland | [Olga.Klimecki@unige.ch](mailto:Olga.Klimecki@unige.ch) |
| Pierre |  | KROLAK-SALMON | MD, PhD | HCL | France | [pierre.krolak-salmon@chu-lyon.fr](mailto:pierre.krolak-salmon@chu-lyon.fr) |
| Elizabeth |  | KUHN | MSc | INSERM | France | [kuhn@cyceron.fr](mailto:kuhn@cyceron.fr) |
| Brigitte |  | LANDEAU | MSc | INSERM | France | [landeau@cyceron.fr](mailto:landeau@cyceron.fr) |
| Julie |  | LEBAHAR | MSc | INSERM | France | [lebahar@cyceron.fr](mailto:lebahar@cyceron.fr) |
| Gwendoline |  | LE DU | MSc | INSERM | France | [ledu@cyceron.fr](mailto:ledu@cyceron.fr) |
| Valérie |  | LEFRANC | BA | INSERM | France | [lefranc@cyceron.fr](mailto:lefranc@cyceron.fr) |
| Antoine |  | LUTZ | PhD | INSERM | France | [antoine.lutz@inserm.fr](mailto:antoine.lutz@inserm.fr) |
| Natalie |  | MARCHANT | PhD | UCL | United Kingdom | [n.marchant@ucl.ac.uk](mailto:n.marchant@ucl.ac.uk) |
| Dix |  | MEIBERTH | MSc | UKK | Germany | [dix.meiberth@uk-koeln.de](mailto:dix.meiberth@uk-koeln.de) |
| Florence |  | MEZENGE | BA | INSERM | France | [mezenge@cyceron.fr](mailto:mezenge@cyceron.fr) |
| Jose-Luis |  | MOLINUEVO | MD, PhD | IDIBAPS | Spain | JLMOLI@clinic.cat |
| Inès |  | MOULINET | MSc | INSERM | France | [moulinet@cyceron.fr](mailto:moulinet@cyceron.fr) |
| Valentin |  | OURRY | MSc | INSERM | France | [ourry@cyceron.fr](mailto:ourry@cyceron.fr) |
| Cassandre |  | PALIX | MSc | INSERM | France | [palix@cyceron.fr](mailto:palix@cyceron.fr) |
| Léo |  | PALY | MSc | INSERM | France | [paly@cyceron.fr](mailto:paly@cyceron.fr) |
| Géraldine |  | POISNEL | PhD | INSERM | France | [poisnel@cyceron.fr](mailto:poisnel@cyceron.fr) |
| Anne |  | QUILLARD | MD | INSERM | France | [anne.quillard@ch-flers.fr](mailto:anne.quillard@ch-flers.fr) |
| Géraldine |  | RAUCHS | PhD | INSERM | France | rauchs@cyceron.fr |
| Stéphane |  | REHEL | MSc | INSERM | France | [rehel@cyceron.fr](mailto:rehel@cyceron.fr) |
| Florence |  | REQUIER | MSc | ULG | Belgium | [florence.requier@uliege.be](mailto:florence.requier@uliege.be) |
| Eric |  | SALMON | MD, PhD | ULG | Belgium | [eric.salmon@uliege.be](mailto:eric.salmon@uliege.be) |
| Raquel |  | SANCHEZ | MD, PhD | IDIBAPS | Spain | [rsanchez@clinic.cat](mailto:rsanchez@clinic.cat) |
| Ann-Katrin |  | SCHILD | PhD | UKK | Germany | [ann-katrin.schild@uk-koeln.de](mailto:ann-katrin.schild@uk-koeln.de) |
| Lena |  | SANNEMANN | MSc | UKK | Germany | [lena.sannemann@uk-koeln.de](mailto:lena.sannemann@uk-koeln.de) |
| Corinne |  | SCHIMMER | MSc | UNICAEN | France | [corinne.schimmer@unicaen.fr](mailto:corinne.schimmer@unicaen.fr) |
| Marco |  | SCHLOSSER | MSc | UCL | United-Kingdom | [marco.schlosser@ucl.ac.uk](mailto:marco.schlosser@ucl.ac.uk) |
| Siya |  | SHERIF | PhD | INSERM | France | [siya.physics@gmail.com](mailto:siya.physics@gmail.com) |
| Edelweiss |  | TOURON | MSc | INSERM | France | [touron@cyceron.fr](mailto:touron@cyceron.fr) |
| Denis |  | VIVIEN | PhD | INSERM | France | [vivien@cyceron.fr](mailto:vivien@cyceron.fr) |
| Patrik |  | VUILLEUMIER | MD | UNIGE | Switzerland | [patrik.vuilleumier@unige.ch](mailto:patrik.vuilleumier@unige.ch) |
| Zuzana |  | WALKER | MD | UCL | United-Kingdom | z.walker@ucl.ac.uk |
| Caitlin |  | WARE | MSc | INSERM | France | caitlin.ware@gmail.com |
| Miranka |  | WIRTH | PhD | DZNE | Germany | miranka.wirth@dzne.de |
| Tim |  | WHITFIELD | MSc | UCL | United-Kingdom | tim.whitfield@ucl.ac.uk |
